# Shaping Green Choices: How Sensory Cues Drive Behavior of Wood-Plastic Composites

**DOI:** 10.3390/bs15030383

**Published:** 2025-03-18

**Authors:** Bicheng Wang, Shun An, Kerun Li

**Affiliations:** Faculty of Innovation and Design, City University of Macau, Macau 999078, China; u23092110312@cityu.edu.mo (B.W.); shunan@cityu.edu.mo (S.A.)

**Keywords:** consumer behavior, emotional response, Stimulus-Organism-Response (S-O-R) model, material perception, wood-plastic composites (WPCs)

## Abstract

By decoding the behavioral mechanisms underlying material perception, this study pioneers a sensory nudging strategy to accelerate the adoption of sustainable materials. This study, grounded in the Stimulus-Organism-Response (S-O-R) model, investigates the factors influencing the purchase intention and loyalty of wood-plastic composites (WPCs), specifically examining the impact of material stimuli, surface treatment processes, product carriers, and environmental stimuli on consumers’ perceptions of uniqueness and emotional responses. A total of 335 valid questionnaires were collected and analyzed using Structural Equation Modelling (SEM), with the results indicating that material stimuli and product stimuli were the strongest predictors of perceived uniqueness and emotional responses. Moreover, emotional response had a significantly stronger effect on purchase intention compared to uniqueness perception. Additionally, material familiarity positively moderated the relationship between emotional response and purchase intention. This study provides theoretical support for the marketing of WPCs, emphasizes the significance of integrating material properties, surface treatments, and usage environments in product design, and suggests new avenues for future research, particularly regarding the relationship between multisensory experiences and consumer behavior.

## 1. Introduction

In the context of global sustainable development, challenges such as climate change, biodiversity loss, and resource depletion have compelled humanity to re-evaluate prevailing production and consumption patterns (Duarte Poblete et al., 2024). The concept of sustainable design, which advocates for the production, use, and disposal of materials in ways that minimize their environmental impact, has driven the search for alternatives to traditional resource-intensive materials (Karana, 2012; Sauerwein et al., 2017). In this context, Wood-Plastic Composites (WPCs) emerge as a promising solution, serving as a novel sustainable material (Manu et al., 2022). WPCs are a new type of composite material composed of granular wood components (e.g., wood pellets or wood flour) and a polymer matrix (Khamtree et al., 2024; Schwarzkopf & Burnard, 2016). The incorporation of natural components can enhance the biodegradation potential of WPCs, though most commercial WPCs use non-biodegradable fossil-based polymers as matrices. In addition, WPCs have properties that are superior to those of a single natural material (Nazrin et al., 2020), making them an ideal choice for sustainable product design.

WPCs are increasingly becoming a preferred alternative to traditional resource-based materials (e.g., wood or petroleum) due to their biodegradability, low cost, and availability (Nurazzi et al., 2021). Their applications span a wide range of fields, including indoor and outdoor furniture, automotive interiors, outdoor recreational infrastructure, packaging, and consumer products (Schwarzkopf & Burnard, 2016). Despite the numerous advantages of WPCs, the scale of their market applications remains significantly smaller than that of natural materials (Maraveas, 2020). In addition to industry-related factors such as material properties, production costs, and manufacturing processes that hinder market adoption (Wilkes et al., 2016), the materials science community has conducted limited research on consumer perceptions and acceptance of materials, leading to the failure of many materials to gain a foothold in the competitive marketplace. Therefore, understanding consumer perceptions and behavioral intentions toward WPCs is crucial for driving their marketability and promoting sustainable design.

While studies have explored the impact of the technical properties and production costs of WPCs on their marketability (Lanzoni et al., 2025; Wilkes et al., 2016), there remains a gap in research regarding consumer behavior and material perceptions. Existing studies have predominantly focused on the physical and chemical properties of materials, often overlooking the emotional experiences and behavioral intentions of consumers toward these materials (Rognoli et al., 2022). Moreover, the emotional experience of materials is a complex phenomenon influenced by various factors (Karana et al., 2015; Schmidt et al., 2025). A review of the literature reveals that most existing studies are dominated by single-variable analyses and lack investigations into the combined effects of multiple factors, such as the material itself, surface treatment processes, products, and environmental stimuli. Therefore, this study aims to address this gap by exploring how these stimuli influence consumers’ perceptions of uniqueness, emotional responses, and ultimately, purchase intention and loyalty toward WPCs, using the Stimulus-Organism-Response (S-O-R) model. The objective of this study is to provide an in-depth analysis of consumers’ behavioral intentions toward WPCs and the underlying mechanisms in the context of sustainable development, thereby offering theoretical support for material design and marketability.

Therefore, this study not only focuses on the technical characteristics of WPCs but also explores consumers’ emotional experiences and behavioral intentions toward them. By introducing the S-O-R model and offering a comprehensive framework for analyzing consumers’ perceptions and reactions to WPCs, this study is the first of its kind in the existing literature. Additionally, this study innovatively integrates the impact of multiple factors related to WPC usage on consumer behavior, rather than focusing solely on a single variable. This integrated multifactor analysis offers new insights into consumer acceptance of WPCs and contributes to promoting their widespread adoption in the marketplace.

The core question of this study is: How do material stimuli, surface treatment stimuli, product stimuli, and environmental stimuli influence consumers’ perceptions of uniqueness and emotional responses to WPCs and, subsequently, their purchase intention and loyalty? To address this question, this study employs the S-O-R model to collect data on consumer perceptions and behaviors by simulating various usage scenarios and product carriers using laboratory-prepared WPC samples, computer-aided design tools (e.g., Adobe Photoshop), and artificial intelligence-generated content (AIGC) technologies. Structural Equation Modelling (SEM) analysis is used to reveal the mechanisms by which each stimulus influences consumer perceptions and behaviors and to propose corresponding design strategies. Furthermore, as research demonstrates that individual experience and culture shape emotion and cognition (Ulrich, 1983), this study also examines the moderating effect of consumers’ familiarity with the material on their purchase intention and loyalty. By understanding how various stimuli influence users’ perceptions and behaviors, designers and materials engineers can support the transition to sustainable production and consumption through the innovative use of resources, materials, and manufacturing processes, thereby enhancing market acceptance. The findings of this study will provide both theoretical support and practical guidance while offering new perspectives on material design and product development. Moreover, this study introduces a novel methodological framework at the intersection of materials science and consumer behavior, grounded in the S-O-R model, to advance the development of sustainable design.

## 2. Research Model and Hypotheses Development

### 2.1. The S-O-R Model

The Stimulus-Organism-Response (S-O-R) model is a well-established framework in environmental psychology (Mehrabian, 1974). The model explains how an external stimulus (S) influences a consumer’s internal mental state (organism), ultimately triggering a behavioral response (R) of approach or avoidance. In recent years, the S-O-R model has been applied across various fields, including marketing, advertising, and online shopping (Chopdar & Balakrishnan, 2020; Meng et al., 2021; Mim et al., 2022). The model is widely recognized for its utility in assessing consumer responses, including evaluating the use of social e-commerce platforms (Hewei & Youngsook, 2022), studying restaurant environment experiences (Kabadayi et al., 2023), and examining the impact of gym service landscape stimuli on fitness center members’ decision-making processes (Özgen et al., 2024). The S-O-R model provides a comprehensive framework for understanding consumer perceptions and attitudes toward external stimuli, as well as their subsequent behaviors (Argyris et al., 2021; B. Lin & Shen, 2023; So et al., 2021). Therefore, building on the traditional S-O-R model, this study seeks to explain changes in the WPCs experience resulting from various stimuli and the impact of these responses on consumer behavior.

The ontology of the S-O-R model is based on the ‘stimulus-response theory,’ which posits that subjects respond after being stimulated by an external object. Accordingly, stimuli are signals that shape the user experience, influence perception, and serve as the starting point for the decision-making process (Nieves-Pavón et al., 2023). To facilitate the large-scale industrial application of WPCs within a sustainable context, physical stimuli can be explored to simulate the material stimuli in real-world usage scenarios. A substantial body of literature has explored the impact of visual and environmental stimuli on user perception and behavior. For instance, Laroche et al. (2022) found that animated images elicited greater feelings of pleasure compared to static images, which, in turn, led to more favorable website attitudes and higher purchase intentions. Similarly, studies on hotel environments have demonstrated that environmental stimuli significantly affect customer loyalty (Jani & Han, 2015).

A comprehensive review of the literature reveals that, while the S-O-R model has been extensively applied to purchasing behavior in various domains, research on the complex relationship between materials, design, and consumption behavior—particularly the perceptual and emotional connections between stimulus application and consumption behavior and loyalty—remains limited. Building on its solid theoretical foundation and empirical support, this study extends the S-O-R framework to explore how various stimuli influence the experience of WPCs and related consumption behavior.

### 2.2. Stimuli and Organism

In consumer environments, stimuli serve as the initiating stage of the consumer’s internal state and subsequent response behavior. The following section examines various potential mechanisms of influence, providing theoretical support for the impact of these stimuli on consumers’ perceptions of uniqueness and the formation of emotional connections.

#### 2.2.1. Material Stimuli

Wilkes et al. experimentally demonstrated a correlation between user perception and the physical properties of the material (Wilkes et al., 2016). Nakamura et al. (1994) further emphasized the influence of the anisotropic characteristics of natural materials on psychological assessments. WPCs, due to their material specificity, consist of a thermoset or thermoplastic polymer matrix, with wood acting as the other main phase. The wood component can vary in shape or size and functions as a filler and/or reinforcement within the composite. Additives comprise a small percentage of the overall composite but are critical to both processing and the final product’s performance (Schwarzkopf & Burnard, 2016). Variations in material ratios in WPCs can significantly influence material perception (Thundathil et al., 2023b). Stark and Cai (2021) support the idea that wood can be combined with non-wood materials to produce composite products with unique properties. Broman (2001) asserts that the random ‘imperfections’ in wood’s material properties contribute to a unique and harmonious emotional experience. WPCs differ from standardized man-made materials in that, while even the smallest imperfection or accident from natural growth can disrupt visual perfection, the material’s uniqueness allows these imperfections to serve as accents (Ashby & Johnson, 2003). This property enables materials to yield unique products even under mass production conditions, thereby triggering a personalized material experience (Karana et al., 2015). Ashby and Johnson (2003) argue that, before a material takes on a recognizable form, its inherent uniqueness is concealed, but if handled appropriately, it can be transmitted to the design. Research has demonstrated that material uniqueness can be achieved through perceived naturalness (Thundathil et al., 2024). In the context of wood-plastic composites (WPCs), visual cues play a pivotal role in naturalness perception by providing initial impressions of material appearance through visible characteristics such as color, texture, and surface patterns. Concurrently, tactile feedback remains indispensable, as users evaluate surface properties—including roughness and thermal sensation—through direct physical interaction.

In this study, wood-plastic composites with different wood flour mixing ratios, wood species, and wood flour particle sizes were selected as internal material variables to investigate the effect of material stimuli on consumers’ internal states. Accordingly, the following hypothesis is proposed:

**H1.**  *Material stimuli have a positive effect on perceived uniqueness.*

Materials evoke a variety of emotions, such as surprise, disgust, disappointment, and curiosity (Y.-T. Chen & Chuang, 2014; Harris et al., 2021; Marschallek & Jacobsen, 2022). Research has shown that the use of wood contributes to a positive psychological impression, evoking feelings of nature, warmth, relaxation, and a desire for use (J. Li et al., 2021). To fully describe the emotional experience of materials, Giaccardi and Karana (2015) defined four levels of experience: the sensory level (e.g., soft or rough), the interpretive level (e.g., modern or nostalgic), the affective level (e.g., surprising or disgusting), and the behavioral level (e.g., inviting to touch). These four levels are interconnected, collectively building the emotional connection associated with the material. Karana (2012) states that WPCs convey a pro-nature emotional connection through visible particles and fibers, natural colors (primarily brown), random patterns, and degradation. Similarly, a material-driven product design study confirmed the ecological advantages of natural materials, positively influencing user emotions (C. Li et al., 2024). Therefore, we argue that materials serve as a medium, enabling consumers to generate an emotional response through interaction. Based on this, the following hypothesis is proposed:

**H2.**  *Material stimuli have a positive effect on emotional responses.*

#### 2.2.2. Surface Treatment Stimuli

Surface treatment can convey information that engages users and creates associative spaces (Ashby & Johnson, 2003). Humans are highly sensitive to subtle changes in surface texture (Skedung et al., 2013) and can detect differences in surface chemistry even when surface roughness remains constant (Carpenter et al., 2018). These signals are particularly important for perceiving variations in surface treatments (Ding et al., 2017). Harris et al. (Harris et al., 2021) demonstrated that the frictional characteristics of material surfaces can be modified by incorporating different matting agents to alter surface texture or by using various resins in topcoats. Their study established a correlation between surface treatment processes and perception. In an experiment investigating non-color material parameters, Wastiels et al. (2012) found that surface gloss and roughness significantly influence the perception of warmth across various materials. By using color-specific virtual samples to filter out color effects, the study highlighted the role of surface texture in shaping sensory perceptions. Additionally, it has been shown that the tactile friction and natural feel of wood can be mimicked using textured polymers by precisely designing surface texture parameters (L. Zhang et al., 2021).

Although previous studies have explored the relationship between surface treatment processes and emotional perception, no research has specifically examined WPCs or the concepts of perceived uniqueness and emotional response in this context. This suggests a limited understanding of how surface treatment processes influence these perceptions, particularly for WPCs. Based on this gap, we propose that when consumers perceive surface treatment processes, they are stimulated to develop uniqueness perceptions and emotional responses:

**H3.**  *Surface treatment stimuli have a positive effect on perceived uniqueness.*

**H4.**  *Surface treatment stimuli have a positive effect on emotional responses.*

#### 2.2.3. Product Stimuli

The user experience of materials within products is crucial, as consumers evaluate and experience materials almost exclusively within a product environment. The influence of the product and its usability environment significantly impacts the material experience (Manu et al., 2022). It is well established that human perception of materials varies depending on the visual environment (Fleming, 2017). The literature indicates that material carriers strategically influence consumer responses to product design by stimulating cognitive and emotional reactions (Rindova & Petkova, 2007). In the context of wood, consumer attitudes differ depending on its application, with wood flooring in interior spaces being perceived as the most habitable (J. Li et al., 2021; Sakuragawa, 2006).

Ashby and Johnson (2003) argue that the shape of a product can distinguish it from similar products within the same price range for identical functions; in other words, the material carrier can create a unique perceptual experience. Similarly, Franke and Schreier (2008) assert that products can enhance the perception of differentiation from other consumers and their possessions. In summary, the product enhances consumers’ perceptions of uniqueness by providing a richer, more authentic, and more complete perceptual experience of the material. Based on this understanding, the following hypothesis is proposed:

**H5.**  *Product stimuli have a positive effect on perceived uniqueness.*

In addition, consumer perceptions are influenced by multiple sensory systems integrated into the appearance of a product (Workman & Caldwell, 2007). On this basis, Y. Lee et al. (2018) suggested that products communicate with consumers through their form and function, potentially resulting in effective emotional connections. The appearance of a product not only affects consumers’ perceptions of features such as its value and worth but also shapes consumer behavior (Heitmann et al., 2020) and fosters emotional connections with consumers (D’Souza et al., 2010; Veryzer & Hutchinson, 1998). According to product semantics theories, all products express themselves through attributes such as shape, form, color, and texture. These attributes communicate with users and are never neutral within their context (Demirbilek & Sener, 2003). For instance, a sports car may evoke associations with power, speed, and a sense of success, leading to perceptions of a confident and ambitious personality. This personality stems from the interplay of morphological attributes, which play a critical role in shaping consumers’ emotional responses to a product (Chowdhury et al., 2015; Sunstrum et al., 2024). Therefore, it can be understood that consumers experience interactions with materials through products, and factors such as the formal appearance and functionality of products make them more susceptible to emotional responses. Based on this understanding, the following hypothesis is proposed:

**H6.**  *Product stimuli have a positive effect on emotional responses.*

#### 2.2.4. Environmental Stimuli

It is an indisputable fact that human perception is influenced by the environment or context (Higuera-Trujillo et al., 2021; Köster & Mojet, 2015). Research has shown that the environment can influence an individual’s consumption behavior by affecting mood, with mood serving as a determining factor in some cases (G. Das & Varshneya, 2017). For instance, in a review study of indoor environments, researchers found that people in such environments were constantly exposed to various indoor environmental stimuli, including thermal, visual, acoustic, and air-quality-related factors, all of which impacted their perceptions and behaviors (Schweiker et al., 2020). Liaw (2007) found that the visual elements of environmental stimuli, such as interior design, visual effects, colors, and aesthetics, significantly influenced customers’ emotions in retail environments. In a comparative study of wood use in interior spaces, J. Li et al. (2021) demonstrated that physical attributes such as the extent of wood usage, wood coverage, and surface variations (e.g., different types of wood and their applications) had a significant effect on the visual psychological responses of individuals within wooden interior spaces.

Building on this, Manu et al. (2022) proposed that the inherent properties of natural materials, when combined with environmental factors, may impart unique properties to bio-composites. Therefore, the design and implementation of environmental stimuli play a crucial role in enhancing consumers’ perception of uniqueness. The creation of a stimulating and enriching environment through such stimuli can help differentiate WPCs from other products. In light of this, we propose the following hypothesis:

**H7.**  *Environmental stimuli have a positive effect on perceived uniqueness.*

Similarly, the interactive connection between the body and various elements of space fosters an emotional, personal connection. In turn, emotional responses reflect emotions and feelings elicited by environmental stimuli (Mehrabian, 1974). Previous research has argued that space can be understood phenomenologically, engaging both the body and environmental stimuli to create emotional connections (K. Lee, 2022). People interact with multiple senses in space (X. Chen et al., 2022), and these sensory experiences are integrated through the body, which continuously interacts with materials and surroundings. This interaction resonates in our consciousness through sensory-bodily spatial experiences, emphasizing the material experiences within the environment. This view is supported by a study on Huizhou wood carvings, where Fang et al. (2024) argued that wood carvings, when placed within spatial environments, transcend their traditional decorative role. Instead, they actively contribute to the production of spatial concepts through narrative imagery, evoking cultural memories, linking socio-cultural contexts with personal emotions, and directly or indirectly influencing others. Based on these considerations, we propose the following hypothesis:

**H8.**  *Environmental stimuli have a positive effect on emotional responses.*

### 2.3. Organism and Response

Organisms contain internal cognitive and affective processes, which serve as intermediate states between stimulus and response in the S-O-R framework (Arora et al., 2020). In order to increase the purchase intention and loyalty of WPCs, this study focuses on the unique emotional experiences that distinguish them from other materials, namely, uniqueness perception and emotional response.

#### 2.3.1. Perceived Uniqueness and Purchase Intention

Perceived uniqueness refers to the extent to which customers perceive a product as different from others in the same category (Tian et al., 2001). According to the Oxford Dictionary, uniqueness is defined as “a very special or unusual quality” or “the fact of being unique in its kind”. Product characteristics not only define the concept of uniqueness but can also be shaped by consumer response. From the consumer’s perspective, a unique product is often perceived as unusual, novel, or unfamiliar (Shi et al., 2020). Unique products tend to evoke positive emotions (Zhou & Nakamoto, 2007) and are often associated with high quality (Chan et al., 2015).

The argument that “perceived uniqueness influences consumers’ future purchase intentions and willingness to spend” has been validated across various domains, including food (Olsen et al., 2021), luxury goods (M. Das et al., 2021), and second-hand goods (Kawulur et al., 2022). For example, in an experiment where consumers designed their own watches, researchers found that participants valued the perceived uniqueness of the product (Franke & Piller, 2004). An alternative explanation for this finding is that participants’ product-related preferences are heterogeneous, and their distinct product solutions reflect these varying preferences. This idea is supported by a study by Franke and Schreier (2008), which suggests that the more unique a consumer perceives a self-designed product to be, in comparison to a standard off-the-shelf product, the more they are willing to pay for it. Based on these findings, we argue that uniqueness perception may lead to positive purchasing behavior. Therefore, we propose the following hypothesis:

**H9.**  *Uniqueness perception has a positive effect on purchase intention.*

#### 2.3.2. Emotional Responses and Purchase Intention

According to emotion-as-information theory, consumers rely on emotions as a key source of information in the decision-making process, with their general feelings driving both cognitive evaluations and subsequent behaviors (Chang & Tuan Pham, 2013). Numerous studies have demonstrated that consumer emotions play a crucial role in purchasing behavior, evaluation, and decision-making processes (Ladhari et al., 2008). The importance of affective responses is further supported by the research of Raghunathan and Pham (1999), who proposed that when consumers are making decisions for themselves, they tend to disregard more objective information and instead rely more heavily on emotional cues. Recent studies have shown a significant positive correlation between affective responses and purchase intention (Kim & Lennon, 2013; C. Li et al., 2024). For instance, a study by Zanger et al. (2022) revealed that consumers’ emotional responses could directly or indirectly increase purchase intention. Similarly, in a study on smart speaker products, it was found that emotion-related responses from users had a significant direct impact on customers’ willingness to purchase smart speakers (Ling et al., 2021). Therefore, it is reasonable to hypothesize that consumers’ emotional responses influence their purchase intentions.

**H10.**  *Emotional response has a positive effect on purchase intention.*

#### 2.3.3. Purchase Intention and Loyalty

Purchase intention refers to the probability that a consumer is willing to adopt a certain purchase behavior. It can be viewed as the subjective tendency of consumers to choose a particular product and has been identified as an important predictor of consumer behavior (Mullet & Karson, 1985). Beyond technical features that fail to meet long-term user satisfaction, it has been argued that products are often discarded because users lose their loyalty to the product (Schifferstein & Zwartkruis-Pelgrim, 2008). Thus, evoking loyalty is not only a potential motivator for driving consumers to purchase WPCs but also a critical consideration for sustainable design (Agost & Vergara, 2020).

Most prior studies have examined both purchase intention and loyalty as outcome variables (K. C. Anderson et al., 2014; Hong & Cho, 2011), exploring the effects of various stimuli on these factors. Additionally, studies on brand loyalty (Sivaram et al., 2019) and shop loyalty (Mainardes & Cardoso, 2019) have highlighted their impact on purchase intention, typically focusing on consumers who are already familiar with similar products or have prior experience with a specific brand. In contrast, loyalty in the context of WPCs emphasizes whether consumers will continue to use or recommend WPCs long after making a purchase or having experience with them. Since loyalty is a key driver in the marketing of WPCs and emotional perceptions and decision-making behaviors associated with WPC use are prerequisites for loyalty, it is expected that purchase intention will positively influence consumer loyalty toward WPCs. Based on this, we propose the following hypothesis:

**H11.**  *Purchase intention has a positive effect on loyalty.*

**Figure 1:**
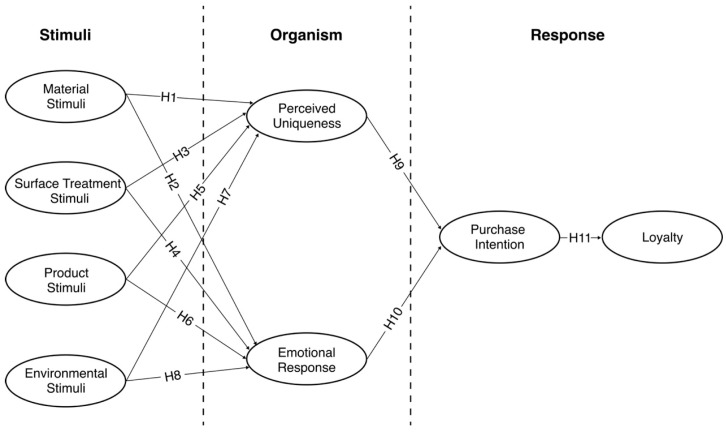
Theoretical model and hypothesis (Model 1).

Building on these hypotheses, we propose a model (Model 1) where material, surface treatment, environment, and product act as stimuli (S); perceived uniqueness and emotional response serve as organisms (O); and purchase intention and loyalty function as response variables (R) (Figure 1).

### 2.4. Moderating Variable

Familiarity is a simple, effective, and widely used method for segmenting consumers (Y. Zhang et al., 2021). In the context of fashion retail brands, brand familiarity has been shown to have both direct and indirect effects on purchase intention (G. Das, 2015). Psychological research indicates that people tend to develop a preference for stimuli with which they are familiar, as repeated exposure increases perceptual fluency—the ease with which the stimulus is processed (Naderi et al., 2020). Therefore, investigating the moderating role of material familiarity can provide academics and practitioners with valuable insights into consumer behavior toward products made with WPCs.

People’s preconceptions or familiarity with a material significantly influence how they experience it (Wilkes et al., 2016). Prior experiences, personal memories, and associations allow participants to infer tactile perceptions through vision (Pallasmaa, 2024). For example, people “know” that wood is warm to the touch and steel is cold, and they reflect these associations in their visual perception of materials. This phenomenon suggests that greater familiarity with a material may make it more difficult for consumers to perceive its uniqueness. Furthermore, an empirical study found that when consumers are unfamiliar with a product, they perceive greater uncertainty and risk when faced with non-consistent attributes due to their lack of experience with the product (Ma et al., 2014). Based on these findings, we hypothesize that while uniqueness perception typically triggers emotional responses and enhances purchase intention, the impact of this perception may be weakened when the product or material becomes overly familiar. Accordingly, we propose the following hypothesis:

**H12.**  *The influence of perceived uniqueness on purchase intention is negatively moderated by material familiarity.*

Familiarity has been shown to enhance consumers’ affective responses, particularly toward materials or brands with which they have already established an emotional connection (Chaudhuri, 2006). For example, in Nordic countries, people tend to have a deeper emotional connection with wooden products due to a long tradition of wooden construction, making them more likely to shop for products containing wood (Mayo, 2015). Similarly, Alba and Hutchinson (1987) suggested that consumers with higher familiarity are more likely to perceive a product positively when exposed to it. Based on this, it is argued that familiarity with a material strengthens consumers’ emotional responses, which in turn enhances their purchase intention:

**H13.**  *The effect of affective response on purchase intention is positively moderated by familiarity with the material.*

**Figure 2:**
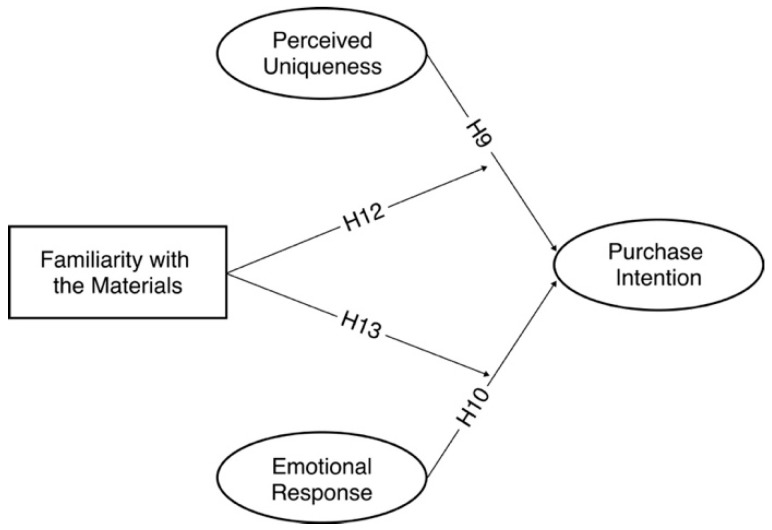
Hypothetical model with familiarity as a moderator (Model 2).

To examine the extent to which familiarity with the material moderates the study’s findings, we propose a moderating variable model (Model 2) (see Figure 2).

## 3. Materials and Method

### 3.1. Stimuli

This study focuses on exploring the effects of various stimuli on consumer perception and behavior. The selection of materials and products in this study aims to simulate positive consumer experiences, thereby validating the market potential of wood-plastic composites (WPCs) within a sustainability context. The stimuli were chosen based on the research on material perception by Karana et al. (2015), ensuring that the variable design aligns with the Stimulus-Organism-Response (S-O-R) model to maintain consistency between theoretical frameworks and empirical findings. To ensure the reliability of the findings, we employ the control variable method (Meyn, 2008) in the experimental design, controlling for factors such as stimulus presentation, experimental environment, and material uniformity, thereby minimizing potential bias or interference. Given the aim of this study to analyze the combined effects of multiple stimuli on sensory responses and consumer behavior towards WPCs, rather than the specific mechanisms through which a single stimulus affects perception, we select a broad range of stimulus variables. These variables encompass different material properties, variations in surface treatment processes, diverse application scenarios, and different product carriers.

**Figure 3:**
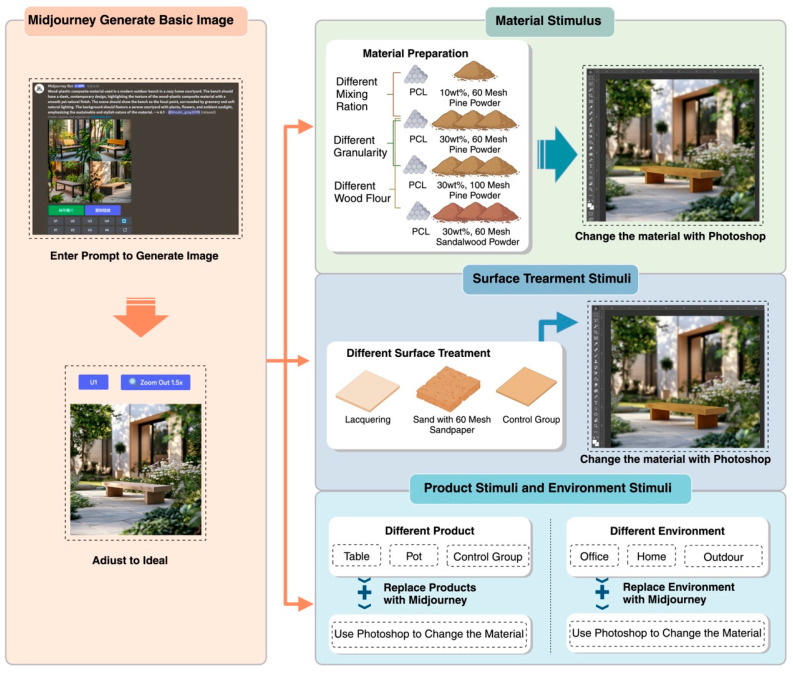
Stimulus Preparation Procedures, Created with Biorender.com (accessed on 13 February 2025).

Furthermore, previous studies have shown a high correlation between on-site environmental evaluations and those obtained through photographs (Stamps, 1990, 2010), with vision generally being more dominant than touch in material perception (Wastiels et al., 2012). Based on these findings, this study uses images to simulate real-life usage scenarios for different stimuli. Specifically, as shown in Figure 3, we use computer-aided design (Adobe Photoshop) in combination with Artificial Intelligence Generated Content (AIGC) technologies (e.g., Midjourney) to synthesize photographs of real WPCs in various contexts. This approach allows participants to engage in the study by viewing these images, enabling a more visual assessment of the effects of different stimuli on perception and behavior.

#### 3.1.1. Preparation of WPCs

**Figure 4:**
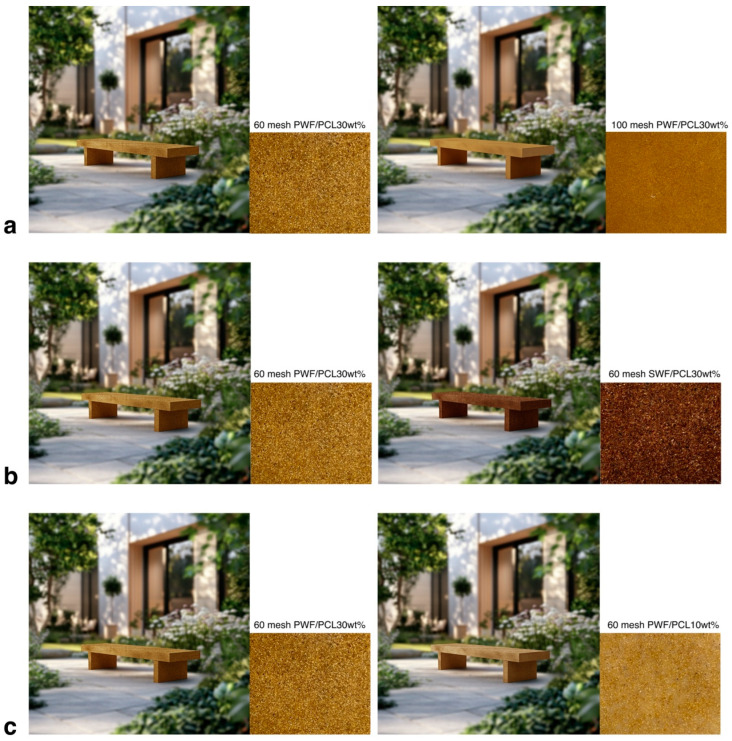
Material stimuli, (**a**) Different wood flour particle sizes; (**b**) Different wood species; (**c**) Different mixing ratios.

In this study, pine wood and sandalwood were selected to prepare wood flour, while polycaprolactone (PCL) was used as the resin matrix to produce WPCs with varying properties. The composites were prepared with two levels of particle size (60 mesh and 100 mesh), two mixing ratios of wood flour and PCL (10 wt% and 30 wt%), and two wood species: pine wood flour (PWF) and sandalwood flour (SWF). Specific details of the material preparation process are provided in Appendix B. After preparation, the materials were placed flat under natural light and photographed using a digital camera to capture their closest real-state appearance. The resulting material images were then processed digitally. Using Midjourney, a courtyard scene was generated, and Adobe Photoshop CC 2019 software was employed to apply the photographed material images onto virtual products. This approach enables the simulation of material stimuli in a consistent scene, showcasing the effect of different materials in the same environment (Figure 4). To ensure that fine material details were recognizable in the Midjourney renders, high-resolution photographs of the WPC samples were used, and close-up views were incorporated where necessary to highlight texture and surface treatments.

#### 3.1.2. Surface Treatment Stimuli

**Figure 5:**
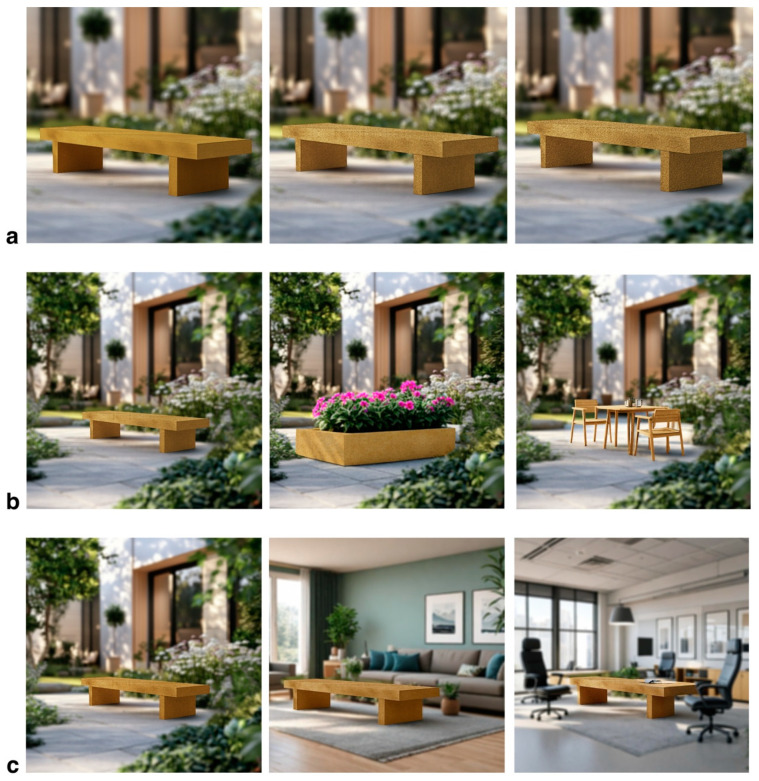
(**a**) Surface Treatment Stimuli; (**b**) Product Stimuli; (**c**) Environment Stimuli.

Three identical WPCs samples (60 mesh, 30 wt% mixing ratio of pine powder and PCL) were selected for surface treatment and numbered as M1, M2, and M3, respectively. Sample M1 underwent a paint treatment, with the surface left to dry. The surface of M2 was sanded using 60 mesh sandpaper, and the dust was subsequently removed. M3 served as the control group, without any surface treatment. After the treatment, the surface conditions of these materials were photographed under natural light. The images were then uploaded to the computer, and Adobe Photoshop was used to combine the photographs of the different surface treatments with a courtyard scene generated by Midjourney. This process simulated the effects of the different surface treatments on the products within the same scene (Figure 5a).

#### 3.1.3. Selection of Products

Three common products used in daily life were selected as material carriers in this study: C1 (table), C2 (flower pot), and C3 (patio seat). These products were chosen to represent both indoor and outdoor usage scenarios, combining decorative and practical functions. C1 (Table) was selected as an example of indoor furniture because tables are common, practical household items suitable for testing the performance of WPCs in daily use (Smardzewski, 2015). C2 (Flower Pot) was chosen as a representative decorative product, serving as a landscape planter (Asante-Kyei, 2018). Finally, C3 (Patio Seat) was selected to demonstrate the material’s performance in an outdoor setting, representing one of the most important application scenarios for WPCs (Oye, 2022). Products C1-C3 were integrated into the patio scene generated through Midjourney, and the material map of sample M3 was applied to their surfaces using Adobe Photoshop. This process resulted in product visuals that depicted different material carriers within the same scene (Figure 5b).

#### 3.1.4. Environmental Stimulus Settings

To comprehensively assess the perceived effects of WPCs across various usage scenarios, three typical environments were selected for this study: the home environment, the work environment, and the outdoor environment. The home environment was chosen as it serves as the central space for daily living activities (Smith, 1994). The work environment represents functional and professional contexts (Rotimi et al., 2024), where consumers interact with materials in a high-frequency and high-demand setting. The outdoor environment, particularly the patio setting, was included due to its relevance as a primary application area for WPCs (Q. Sun et al., 2020). Using Midjourney, images of these three environments were generated, with the C3 product being incorporated into each scene. Adobe Photoshop was then employed to apply the M3 material to the product’s surface. This process resulted in visual representations of the products within various environmental settings, all featuring the same material and surface treatment (Figure 5c).

### 3.2. Data Collection

In this study, standardized data were collected through a questionnaire to test the research hypotheses. Before conducting the formal survey, a pretest was conducted to evaluate the questionnaire’s validity and operability, with 25 participants engaged in the process. During the questionnaire design phase, expert reviews were combined with focus group discussions, and minor adjustments were made to the questionnaire based on feedback from experts and group members. The results of the pre-survey indicated that the Cronbach’s alpha values for all scale dimensions exceeded 0.7, demonstrating strong internal consistency.

The formal questionnaire comprised three thematic sections and a basic information section. The basic information section included demographic questions (age, gender, and occupation) and assessed participants’ familiarity with WPCs. The first thematic section focused on questions related to stimuli (material, surface treatment, products, and environments). The second section addressed participants’ perceived uniqueness and emotional responses to WPCs. The third section explored questions about purchase intentions and loyalty. All questions, except those related to demographics, were measured using a 7-point Likert scale (Likert, 1932) (1 = completely disagree, 7 = completely agree). The questions were carefully selected to ensure scientific validity and applicability. Appendix A provides a detailed list of the scales and items used in this study, along with their sources. These scales were adapted and developed with reference to existing literature during the design process.

### 3.3. Subjects

This study was conducted in strict compliance with the ethical principles outlined in the Declaration of Helsinki (2024). All participants provided informed consent before participating, either in writing or electronically. Data collection took place between November 2024 and January 2025. The study employed a questionnaire-based approach for data collection. Questionnaires were distributed both offline (primarily at the University of M) and through online social media platforms. All participants were informed about the purpose of the survey, the responsible organization, and the guarantees of anonymity and data confidentiality. Potential respondents were politely invited to participate, and questionnaires were distributed only to those who agreed, ensuring the reliability and accuracy of the results. For sections of the questionnaire involving stimuli, participants were required to observe the relevant pictures before completing their responses. A total of 379 questionnaires were collected, including 219 online and 160 offline submissions. After screening, 335 valid questionnaires were retained for analysis.

### 3.4. Data Analysis

The data in this study were analyzed using SPSS 28 and AMOS 26.0. To test the hypotheses proposed in Model 1, the two-step structural equation modeling (SEM) approach suggested by J. C. Anderson and Gerbing (1988) was employed. The process involved the following steps: First, the validity and reliability of the scales were evaluated through confirmatory factor analysis (CFA). Next, the structural model was assessed, and the hypothesized model was validated using SEM. To evaluate the fit of the model to the data, the following fit indices were calculated: the chi-square (χ^2^), the comparative fit index (CFI), the goodness-of-fit index (GFI), and the normative fit index (NFI). A value close to 0.9 or 1.0 for these indices indicates a well-fitted model. Additionally, the root mean square error of approximation (RMSEA) was calculated, with values preferably less than 0.05 to ensure the robustness of the model. In addition to Model 1, the study constructed Model 2 to test Hypotheses 12 and 13, incorporating material familiarity as a moderating variable in the analysis.

## 4. Results

### 4.1. Measurement Model Assessment

In this study, unidimensionality, reliability, and construct validity were assessed for all latent variables, including material stimuli, surface treatment stimuli, product stimuli, environmental stimuli, perceived uniqueness, emotional response, purchase intention, and loyalty. As shown in Table 1, the confirmatory factor analysis (CFA) results indicate a good model fit to the data (χ^2^ = 406.993, df = 224, χ^2^/df = 1.817, GFI = 0.908, CFI = 0.970, NFI = 0.936, IFI = 0.970, RMSEA = 0.049). All items load above 0.60 on their corresponding factors and are significantly associated with the specified constructs (*p* < 0.01), validating the unidimensionality of each scale. To address the potential issue of common method bias (CMB), all variables, measured using common instruments across scales, were examined using the Harman single-factor test, which is widely employed to assess common measurement biases (Nieves-Pavón et al., 2023). In the single-factor test, all indicators were subjected to an exploratory factor analysis (EFA) to determine whether a single factor explained most of the variance or if the first factor explained more than 50% of the variance (Chopdar & Balakrishnan, 2020). The results show that the first factor accounts for 34.779% of the variance, indicating the absence of common method bias. The reliability of the study constructs (Cronbach, 1951), along with the composite reliability (which indicates the internal consistency of multiple indicators for each construct), ranges between 0.812 and 0.965, well above the standard requirement of 0.70. Furthermore, the average variance extracted (AVE) values range from 0.812 to 0.917, exceeding the recommended threshold of 0.50 (Fornell & Larcker, 1981), thus validating the convergent validity of the constructs.

Table 2 presents the square root of the AVE, represented by the diagonal values in the correlation matrix for all constructs. Sufficient discriminant validity is established when the square root of each construct’s AVE is greater than its corresponding correlations with other constructs (Fornell & Larcker, 1981). Based on these results, the measurement model demonstrates sufficient reliability and validity. In summary, the tests conducted confirm that the measurement model exhibits strong reliability and validity, supporting the robustness of the proposed model.

### 4.2. Structural Model Evaluation

**Figure 6:**
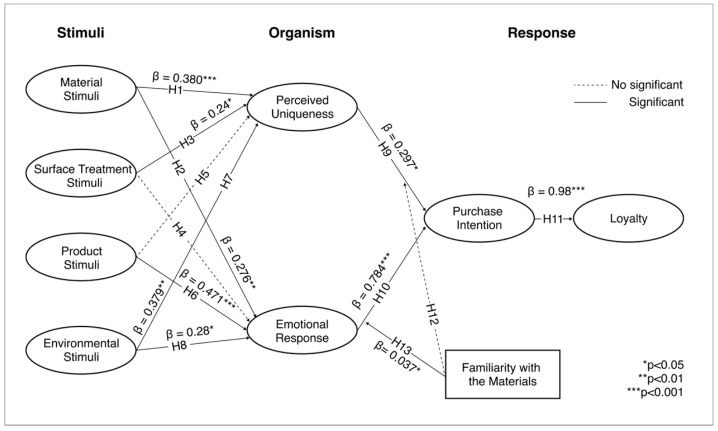
SEM results.

The structural model demonstrates an acceptable fit to the data (χ^2^ = 406.993, df = 224, χ^2^/df = 1.817, GFI = 0.908, CFI = 0.970, NFI = 0.936, IFI = 0.970; RMSEA = 0.049). As shown in Figure 6, most of the structural coefficients are significant at various levels. The relationship between material stimuli and both perceived uniqueness (β = 0.380; *p* < 0.001) and emotional response (β = 0.276; *p* < 0.01) is statistically significant, supporting H1 and H2. Regarding the surface treatment stimuli, the results indicate a significant effect on perceived uniqueness (β = 0.240; *p* < 0.05), but no significant effect on emotional response (β = −0.042; *p* > 0.05), which supports H3 and negates H4. For product stimuli, a highly significant effect is observed on emotional response (β = 0.471; *p* < 0.001), but no effect on perceived uniqueness (β = −0.012; *p* > 0.05), confirming H6 and rejecting H5. The results also show that environmental stimuli significantly influence both perceived uniqueness (β = 0.347; *p* < 0.05) and emotional response (β = 0.280; *p* < 0.05), supporting both H7 and H8. Additionally, perceived uniqueness has a significant positive effect on purchase intention (β = 0.297; *p* < 0.05), and emotional response has a stronger, highly significant effect on purchase intention (β = 0.784; *p* < 0.001), which supports H9 and H10. Finally, purchase intention has an extremely significant positive effect on loyalty (β = 0.980; *p* < 0.001). The coefficients of determination (R^2^) for perceived uniqueness, emotional response, purchase intention, and loyalty are 0.865, 0.873, 0.872, and 0.869, respectively, indicating strong explanatory power in the model.

### 4.3. Analyzing the Moderating Role of Familiarity with Materials (Model 2)

**Figure 7:**
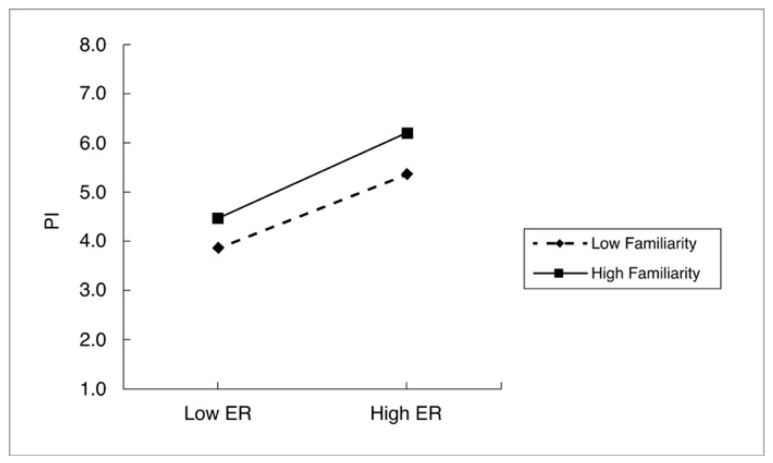
Interaction effect of familiarity X Emotional Response (ER) on Purchase Intention (PI).

Hypotheses H12 and H13 examined the moderating role of familiarity with materials in the relationships outlined in H9 and H10. The moderating effect was assessed by testing the significance of the product term (independent variable X moderator) in the model. The results revealed that familiarity with the material significantly enhanced the relationship between emotional response and consumer purchase intention (β = 0.037; *p* < 0.05), indicating that familiarity with the material strengthens the positive effect of emotional responses on purchase intention. This finding supports H13, with the moderating effect visually represented in Figure 7. However, familiarity with the material did not significantly affect the relationship between perceived uniqueness and purchase intention (β = 0.036; *p* > 0.05), leading to the rejection of H12.

### 4.4. Analysing the Direct and Indirect Effect

To further investigate the total, direct, and indirect effects between perceived uniqueness, emotional response, and loyalty, we conducted SEM in AMOS and bootstrapped the sample for 5000 iterations (Henseler et al., 2016). Table 3 presents the effect model at the 95% significance level. The results show that purchase intention mediates the effects of both perceived uniqueness (β = 0.605, *p* < 0.05) and emotional response (β = 0.639, *p* < 0.001) on loyalty. Although the direct effect was not significant, Hayes and Scharkow (2013) suggested that an indirect effect can still exist even when the direct effect is not significant. Given that the indirect effect of purchase intention was significant, this indicates an indirect, rather than a direct, influence between perceived uniqueness, emotional response, and loyalty. The possible reasons for this phenomenon will be explored in the discussion section.

## 5. Discussion

This study contributes to filling a gap in the existing literature by applying the S-O-R model to WPCs and providing a comprehensive examination of the effects of various stimuli—material stimuli, surface treatment stimuli, product stimuli, and environmental stimuli—on consumers’ perceived uniqueness, emotional responses, and purchase intentions and loyalty. The results demonstrate the relevance of incorporating these stimuli and their associated responses in understanding consumer behavior in the context of WPCs. Perceived uniqueness and emotional response, as key elements of the organism in the S-O-R framework, are found to significantly influence purchase intention and loyalty. By analyzing the stimuli and responses involved in the consumer decision-making process, this study provides insights into how these factors drive consumer loyalty to WPCs products. This finding underscores the importance for designers, materials engineers, and researchers in the field of materials design to adopt a holistic approach when considering the effects of material, surface treatment, product, and environmental contexts on consumers’ perceptual and emotional responses. Such an approach will contribute to a better understanding of consumer behavior patterns and the development of effective strategies for designing and marketing WPC-based products.

Specifically, the study reveals that material stimuli have the most significant effect on perceived uniqueness and are also strong predictors of emotional responses. This suggests that by adjusting the material properties of WPCs—such as the mixing ratio, particle size of wood flour, and type of composite material—consumers’ perception of uniqueness can be effectively stimulated. Moreover, the material properties of WPCs can evoke a wide range of emotional responses, further enhancing consumers’ emotional connections to these products. This finding aligns with Appraisal Theory, which posits that emotions are elicited by individuals’ cognitive appraisals of stimuli (Roseman & Smith, 2001). These findings not only contribute to the existing literature but also align with the research of Rognoli et al. (2011), who suggest that designers can integrate material associations and emotions to develop more appealing and emotionally engaging products.

However, the surface treatment, while significantly influencing perceived uniqueness, was not a significant predictor of emotional response. This finding aligns with the results of Favier et al. (2024) but contrasts with the conclusions of Faucheu et al. (2019). This suggests that surface treatment can convey information and enhance the perceived differentiation of materials, thereby stimulating consumers’ perception of uniqueness. However, users do not appear to specifically associate surface treatment with their emotional responses. One possible explanation for this is that the subtle changes induced by surface treatments may not be easily captured through visual perception alone. Multi-sensory experiences, such as tactile sensations, might be more effective at triggering emotional responses (Spence & Gallace, 2011). Furthermore, the impact of surface treatment on perception may be more pronounced at the sensory level (e.g., uniqueness perception) rather than at the emotional level. This could explain why much of the related research has predominantly focused on material sensory properties (Skedung et al., 2018, 2020).

Product stimuli was found to have the most significant influence on emotional responses, consistent with the findings of Meiselman (2015), thereby validating the hypothesis that products can evoke strong emotional reactions. This confirms that consumers experience significant affective changes in certain contexts, as products, according to the theory of embodied cognition, create interactions that engage both the body and emotions (Zhu & Jung, 2024). However, product stimuli did not significantly influence perceived uniqueness, highlighting that in the context of WPCs, product-related stimuli are not directly linked to perceptions of uniqueness. While other studies suggest that unique products can enhance differentiation among consumers and their possessions (Franke & Schreier, 2008), the lack of significance here may be attributed to consumers’ greater focus on the material properties rather than the product itself. Additionally, the experimental product types may not have sufficiently showcased the uniqueness of the materials, or consumers may have prioritized the functional aspects of the products over their sensory properties (Krause et al., 2023). Regarding environmental stimuli, the findings reveal a positive relationship between environmental stimuli and both perceived uniqueness and emotional response. This aligns with previous research (Fiore et al., 2000; Franz, 2006; Schreuder et al., 2016), supporting the idea that the physical environment plays a critical role in shaping consumer impressions (Bitner, 1992). As I. Y. Lin (2004) observed, visual elements of the environment, such as color, space, function, and lighting, significantly influence emotions and behaviors, further emphasizing the importance of environmental context in consumer experience formation.

In terms of the relationship between perceived uniqueness, emotional response, and purchase intention, it was found that both perceived uniqueness and emotional response had a positive and significant effect on purchase intention, but the effect of emotional response was more significant. This suggests that consumers with higher uniqueness perceptions and more intense emotional responses to WPCs are more likely to form purchase intentions. This result aligns with the study by He et al. (2023), which found that positive emotions have the greatest effect on purchase intention. In addition, there is a positive relationship between purchase intention and loyalty. This finding emphasizes the need to stimulate consumers’ perceptual and emotional responses, as well as their willingness to buy when promoting WPCs, which ultimately fosters loyalty.

Research on the mediating effect of purchase intention suggests that consumers are influenced by external factors when making purchasing decisions, and these factors subsequently influence their internal perceptions and emotional attitudes, which in turn affect their purchase intention, ultimately positively influencing loyalty. However, perceived uniqueness and emotional response do not have a significant direct effect on loyalty but rather exert an indirect effect through factors such as purchase intention. This may be due to the fact that the effects of perceived uniqueness and emotional response are short-term and have a weaker impact on long-term loyalty, or other moderating variables or mechanisms may need to be considered. Therefore, this study provides new perspectives for academics and practitioners by demonstrating that perceived uniqueness and emotional responses can promote loyalty by establishing positive purchase intentions.

Regarding the moderating effect of material familiarity, it was found that familiarity did not have a significant effect on the relationship between perceived uniqueness and purchase intention, which aligns with the study by Thundathil et al. (2023a). The reason for this may be that subjective evaluations based on emotions are influenced by individual preferences, whereas material perceptions are not (M. Sun et al., 2020). However, for consumers more familiar with WPCs, the positive effect of emotional responses on purchase intention was stronger. This implies that familiarity may enhance the emotional connection between the consumer and the material, thereby promoting purchase behavior. This finding extends the study by Calvo Porral and Levy-Mangin (2016) and offers important insights into marketing and promotion strategies for WPCs, particularly in terms of eliciting emotional responses from consumers.

## 6. Conclusions

This study provides valuable insights into consumer behavior regarding WPCs, particularly regarding the drivers of purchase intention and loyalty, through using the S-O-R framework. The study identifies and analyzes various influences, such as material stimuli, surface treatment stimuli, product stimuli, and environmental stimuli, demonstrating their respective roles in shaping both perceived uniqueness, emotional response, purchase intention, and loyalty. The results showed that material stimuli were the strongest predictor of perceived uniqueness, while product stimuli had the most significant effect on emotional response. In addition, material familiarity emerged as a significant moderating factor, influencing the relationship between consumers’ emotional response and purchase intention. These findings not only offer new perspectives to the WPC literature but also highlight the important roles of perceived uniqueness and emotional response in driving consumer behavior, further illuminating their impact on purchase intention and loyalty.

Emotional response is identified as a key variable in predicting consumer purchase intention and loyalty. Another significant contribution of this study is the identification of the moderating role of material familiarity within the research model, where familiarity was shown to significantly moderate the interaction between emotional response (organism) and purchase intention (response). Furthermore, the study explored the role of purchase intention as a mediating variable, refining the understanding of the causal relationship between perceived uniqueness, emotional response, and loyalty, thus providing more nuanced insights into the field.

This study provides a significant theoretical framework for the design and promotion of WPCs. By uncovering the effects of material properties, surface treatment, products, and environmental stimuli on consumer behavior, it offers specific strategic recommendations for designers and manufacturers. Building on these findings, the uniqueness and appeal of products can be enhanced by optimizing material textures, such as introducing biomimetic wood grain patterns or adjusting the composition ratios of composite materials. This approach is highly relevant to consumers’ sensitivity to material stimuli. Furthermore, given the influence of product stimuli on emotional responses, marketers can leverage context-specific promotional strategies—for instance, positioning wood-plastic composites (WPCs) as sustainable options for outdoor leisure applications—to evoke positive emotional reactions among consumers.

Moreover, this study emphasizes the moderating role of material familiarity in shaping consumer behavior. Consumers who are more familiar with WPCs are more likely to form purchase intentions driven by emotional responses. Therefore, policymakers can enhance consumer awareness of wood-plastic composites (WPCs) through subsidies or educational initiatives, leveraging their sustainability advantages to further drive market acceptance. This finding offers a fresh perspective on the marketing and commercialization of WPCs.

Although this study provides valuable contributions, it has certain limitations. First, the cross-sectional study design restricts the ability to infer causality. Future research could adopt a longitudinal design or experimental approach to better capture perceived changes in WPCs and their long-term effects on consumer behavior. Second, the model can be extended with variables like consumer personality and cultural background to improve predictive power. It currently overlooks individual differences (e.g., environmental consciousness) and cultural factors (e.g., collectivism), which future research could address to enhance explanatory power. What’s more, Limited by the data and research design, this study analyzes affective response as a holistic construct that is not broken down into specific sub-dimensions. While this approach is effective in capturing the overall impact of affect, future research could deepen the analysis through more granular dimensions (e.g., aesthetic appeal or sustainability affinity). Lastly, the material perception experiments in this study relied solely on visual stimuli. Although images effectively convey the visual properties of WPCs, they fail to replicate the tactile experience, which is critical for a comprehensive understanding of material naturalness. Future research could consider experimental designs that incorporate multimodal perception (e.g., tactile or olfactory stimuli) to provide a more comprehensive understanding of consumers’ perceptual experiences. Additionally, leveraging augmented reality (AR) and virtual reality (VR) technologies could further enhance these experiments by simulating immersive environments that engage multiple senses, offering a more realistic and dynamic consumer experience.

## Figures and Tables

**Table 1:** Measurement model results.

Construct	Items	Mean (SD)	Loadings	AVE	Composite Reliability
Material Stimuli (MS)	MS1	4.98 (1.59)	0.852 ***	0.685	0.866
	MS2	5.18 (1.44)	0.815 ***		
	MS3	5.05 (1.52)	0.815 ***		
Surface Treatment Stimuli (ST)	ST1	5.3 (1.39)	0.820 ***	0.644	0.844
	ST2	5.35 (1.37)	0.797 ***		
	ST3	5.2 (1.4)	0.791 ***		
Product Stimuli (PS)	PS1	5.59 (1.24)	0.792 ***	0.601	0.82
	PS2	5.43 (1.32)	0.745 ***		
	PS3	5.28 (1.37)	0.788 ***		
Environmental Stimuli (ES)	ES1	5.36 (1.36)	0.762 ***	0.591	0.812
	ES2	5.42 (1.34)	0.772 ***		
	ES3	5.33 (1.48)	0.773 ***		
Perceived Uniqueness (PU)	PU1	5.32 (1.28)	0.738 ***	0.630	0.833
	PU2	5.12 (1.34)	0.812 ***		
	PU3	5.01 (1.46)	0.828 ***		
Emotional Response (ER)	ER1	5.44 (1.47)	0.787 ***	0.671	0.857
	ER2	5.12 (1.52)	0.834 ***		
	ER3	4.98 (1.54)	0.836 ***		
Purchase Intention (PI)	PI1	5.08 (1.48)	0.824 ***	0.641	0.834
	PI2	4.82 (1.59)	0.872 ***		
	PI3	5.41 (1.32)	0.695 ***		
Loyalty (LY)	LY1	4.86 (1.6)	0.891 ***	0.788	0.917
	LY2	4.9 (1.64)	0.894 ***		
	LY3	4.93 (1.7)	0.878 ***		

Confirmatory factor analysis fit indices: χ^2^/df = 1.817 (Ideal < 3.0); GFI = 0.908 (Ideal > 0.900); CFI = 0.970 (Ideal > 0.900); NFI = 0.936 (Ideal > 0.900); IFI = 0.970 (Ideal > 0.900); RMSEA = 0.049 (Ideal < 0.05). *** Denotes values significant at 95% confidence level. Notes: MS = Material Stimuli; ST = Surface Treatment Stimuli; PS = Product Stimuli; ES = Environmental Stimuli; PU = Perceived Uniqueness; ER = Emotional Response; PI = Purchase Intention; LY = Loyalty.

**Table 2:** Discriminant validity and descriptive statistics of measures.

	MS	ST	PS	ES	PU	ER	PI	LY
MS	*0.828*							
ST	0.703	*0.803*						
PS	0.711	0.694	*0.775*					
ES	0.665	0.726	0.697	*0.769*				
PU	0.751	0.749	0.694	0.720	*0.794*			
ER	0.736	0.665	0.750	0.692	0.749	*0.819*		
PI	0.693	0.677	0.693	0.694	0.754	0.800	*0.801*	
LY	0.628	0.602	0.627	0.622	0.673	0.752	0.800	*0.888*

The diagonal value represents √_AVE_; and the off-diagonal values represent inter-construct correlations for respective variables. Notes: MS = Material Stimuli; ST = Surface Treatment Stimuli; PS = Product Stimuli; ES = Environmental Stimuli; PU = Perceived Uniqueness; ER = Emotional Response; PI = Purchase Intention; LY = Loyalty.

**Table 3:** Mediation effect.

Effects	Effects of PU on LY Mediated Through PI	Effects of ER on LY Mediated Through PI
Total effect	0.319 *^ns^*	0.756 ***
(lower bound, upper bound)	(−0.120, 0.749)	(0.491, 1.077)
Direct effect	−0.285 *^ns^*	0.117 *^ns^*
(lower bound, upper bound)	(−1.162, 0.153)	(−0.447, 0.478)
Indirect effect	0.615 **	0.639 ***
(lower bound, upper bound)	(0.201, 1.471)	(0.298, 1.283)

*** *p* < 0.001, ** *p* < 0.01, *^ns^* Not significant (*p* > 0.05). Note: PU = Perceived Uniqueness; ER = Emotional Response; PI = Purchase Intention; LY = Loyalty.

**Figure A1:**
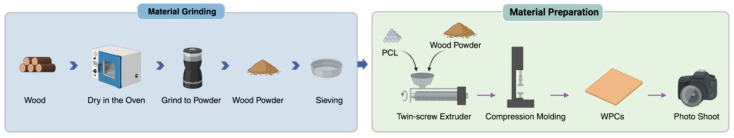
Material Preparation Methods, Created with Biorender.com (accessed on 13 February 2025).

## Data Availability

Data is available upon reasonable request.

## Abbreviations

The following abbreviations are used in this manuscript:

WPCs	Wood-Plastic Composites
PCL	poly-ε-caprolactone
MS	Material Stimuli
ST	Surface Treatment Stimuli
PS	Product Stimuli
ES	Environmental Stimuli
PU	Perceived Uniqueness
ER	Emotional Response
PI	Purchase Intention
LY	Loyalty
SOR	Stimulus-Organism-Response

## Appendix A

**Table A1:** Constructs, indicators, and sources.

Construct	Items	Sources
Familiarity	I am very familiar with wood-plastic composites.	
Material Stimuli	Wood-plastic composites stimulate multiple sensory experiences for me.	(Gao & Lan, 2020)
	The sensory characteristics of wood-plastic composites are diverse and rich.	
	The sensory performance of wood-plastic composites is appealing to me.	
Surface Treatment Stimuli	Surface treatment enhances the sensory experience of the material.	(Desmet & Hekkert, 2007; Peck & Childers, 2003)
	Surface treatment enhances the tactile appeal of wood-plastic composites.	
	Surface treatment enhances the visual appeal of wood-plastic composites.	
Product Stimuli	Wood-plastic composites perform well in various products.	(Sweeney & Soutar, 2001)
	I find wood-plastic composites acceptable in different types of products.	
	Products made of wood-plastic composites meet my quality standards.	
Environmental Stimuli	Environmental factors enhance my overall sensory experience of wood-plastic composites.	(Naderi et al., 2020; Spence et al., 2014)
	Environmental layout influences how I feel about the product/material.	
	The environment makes wood-plastic composites appear more attractive.	
Perceived Uniqueness	Wood-plastic composites are significantly different from other materials in terms of sensory perception.	(Gao & Lan, 2020)
	Wood-plastic composites stand out in their sensory characteristics.	
	Wood-plastic composites can distinguish themselves from other materials in sensory perception.	
Emotional Response	Wood-plastic composites make me feel a connection to nature.	(Park et al., 2010; Spinelli et al., 2014)
	Wood-plastic composites evoke positive emotions in me.	
	I have an emotional connection to wood-plastic composites.	
Purchase Intention	I am likely to purchase products made from wood-plastic composites.	(Waheed et al., 2018)
	Compared to other materials, I am more willing to buy products made from wood-plastic composites.	
	I am open to purchasing products made from wood-plastic composites.	
Loyalty	I tend to use wood-plastic composites.	(Martínez & Del Bosque, 2013)
	I would repurchase wood-plastic composites if needed.	
	I would recommend wood-plastic composites to others.	

## Appendix B

Pine and sandalwood were sourced from Shantou, while poly-ε-caprolactone (PCL) was procured from Shanghai McLean Biochemical Technology Co, Shanghai, China. Pine and sandalwood was washed, cut into uniform pieces, and dried at 120 °C for 24 h. It was then ground in a ball mill for 40 s. The powder was sieved to obtain powders with 60 and 100 mesh, and dried further at 120 °C until constant weight. No surface treatment was carried out on the straw powder, as the aim was to reduce the environmental impact by not using any processes or adding any chemical products. The powder was mixed with PCL in a high-speed mixer, and then melted and mixed for 5–10 min at 175 °C using a twin-roll mill to prepare composites with different wood flour contents, as shown in Figure A1. Finally, it was compression molded at 185 °C for 5 min.
